# Touching! An Augmented Reality System for Unveiling Face Topography in Very Young Children

**DOI:** 10.3389/fnhum.2019.00189

**Published:** 2019-06-11

**Authors:** Michiko Miyazaki, Tomohisa Asai, Ryoko Mugitani

**Affiliations:** ^1^Department of Social Information Studies, Otsuma Women’s University, Tokyo, Japan; ^2^NTT Communication Science Laboratories, Atsugi, Japan; ^3^Advanced Telecommunications Research Institute International (ATR), Kyoto, Japan; ^4^The Faculty of Integrated Arts and Social Sciences, Japan Women’s University, Kanagawa, Japan

**Keywords:** psychology, development, body image, augmented reality, face, body topography

## Abstract

Developmental body topography, particularly of the face, is a fundamental research topic in the current decade. However, empirical investigation of this topic for very young children faces a number of difficulties related to the task requirements and technical procedures. In this study, we developed a new task to study the spatially-sensed position of facial parts in a self-face recognition task for 2.5- and 3.5-year-old children. Using the technique of augmented reality (AR) and 3D face tracking technology, we presented participants with their projected self-image on a screen, accompanied by a digital mark located on parts of their face. We prepared a cheerful visual and auditory reward on the screen when participants showed correct localization of the mark. We then tested whether they could indicate the position of the mark on their own faces and remain motivated for task repetition. To assess the efficacy of this task, 31 2.5- and 11 3.5-year-old children participated in this study. About half of the 2.5-year-olds and 80% of the 3.5-year-olds could perform more than 30 trials. Our new task, then, was to maintain young children’s motivation for task repetition using the cheerful visual and auditory reward. The analysis of localization errors suggested the uniqueness of spatial knowledge of self-face in young children. The efficacy of this new task for studying the development of body image has been confirmed.

## Introduction

Children begin to learn about their own bodies from early in life. They learn several methods for body representation and organize these representations among various modalities, including names of body parts (semantic or conceptual), body topography (spatial or structural), and body schema (somatosensory or controllability; Schwoebel and Coslett, 2005). Several interesting behaviors that derive from the immature emergence or organization of body representations are observed in young children. For example, from the age of 2 years, children draw “tadpole humans” that typically consist of circles with some facial features, representing the head as well as the body (Freeman, 1975; Cox, 2013). Another interesting behavior is called the scale error (Deloache et al., 2004), whereby, after playing with a body-sized large toy car, young children aged around 2 years may attempt to enter and drive a miniature toy car ignoring their body size. Yet another interesting exploration error is the rear-search error (Miyazaki and Hiraki, 2009), which occurs in children aged around 2 years during body part localization of their mirrored self-body. In Miyazaki and Hiraki’s experiment, participants initially attempted to localize a target on the rear of their heads, even though it was placed on their forehead, an error that was observed in over one-third of the 2-year-old participants. These behaviors suggest that young children may have specific and immature body representation(s).

Beyond these observations, it is generally challenging to empirically examine young children’s immature body representations. Compared to adults, children: (1) cannot fully understand verbal instructions; (2) cannot keep themselves motivated; and therefore (3) cannot repeat trials. Since it is necessary to describe the developmental trajectory and differences in body representation between young children and adults, it is useful to seek a solution to the aforementioned difficulties. Although we aimed to explore the development of whole-body representation(s) for future, in this study, as a first step, we developed a new task for evaluating the emergence of face topography in young children, using an augmented reality (AR)-based procedure.

Several previous studies have examined the development of body topography in young childhood (Witt et al., 1990; Brownell et al., 2010, 2012; Camões-Costa et al., 2011; Herold and Akhtar, 2014; Waugh and Brownell, 2015). For example, Brownell et al. (2010) examined the body topography of 20- and 30-month-olds using a sticker placing task. An experimenter demonstrated the task to the children by placing stickers on another experimenter’s specific body parts. The children were then asked to place a sticker on an unnamed body location on themselves. The sticker task began on the nose, then proceeded to include 12 body locations (nose, hand, foot, head, back, neck, forehead, wrist, elbow, calf, temple, and nape). The results revealed an immature body structural knowledge. On average, 20-month-olds were able to locate only two or three of the locations, while 30-month-olds (2.5-year-olds) were able to locate four or five (Brownell et al., 2010). Herold and Akhtar (2014) examined body structural knowledge using a similar sticker task in 2.5-year-olds. They demonstrated the task to the children by placing a sticker on a life-sized drawing of a child. The children were then asked to place a sticker on four body parts (hair, stomach, arm, and foot). The results demonstrated that 16 out of 48 children correctly placed the sticker more than three times (Herold and Akhtar, 2014).

Based on the findings of these previous studies, young children have little knowledge of their own body topography. However, it is possible that children’s knowledge of own body is underestimated. As mentioned above, verbal instruction might be tricky for young children and the task difficulty and complexity could keep children from revealing their potential knowledge about their body structure. That is, they might have an implicit body topography without having any explicit knowledge. In Brownell et al.’s (2010) study described above, after demonstrating the task with another experimenter, children were given the following verbal instruction: “Now you put your sticker on you right there, so it’s just like [name]. You put your sticker right there on you.” This phrasing may be slightly difficult and complex for young children because it requires referential and conceptual inference from the phrases “right there” and “just like [name].” Furthermore, this task required stickers to be placed 12 times and too many repetitions is likely to be boring for young children (Brownell et al., 2010). In our pilot examination for this study, we found that young children were bored by task repetition without any reward. Thus, a new task for evaluating body structural knowledge requires a simple task rule and exciting feedback in order to ensure that children remain motivated for task repetition.

To overcome the problems mentioned above, using the technique of AR and 3D tracking technology, we presented participants with their projected self-image on a screen with one of several famous cartoon characters (digital images). The cartoon character then appeared on various parts of the children’s bodies, and we tested whether the participants could demonstrate correct localization by touching the same parts of their own bodies. We also tested whether they would remain motivated throughout the task despite the repetition.

In this study, as the first step before exploring whole body topography, we developed a new task for evaluating face topography in children aged 2.5 and 3.5 years. Previous studies examining body topography in adult participants have used several perspectives for the estimation: face (Fuentes et al., 2013b; Serino et al., 2015; Estudillo and Bindemann, 2017; Mora et al., 2018; Porciello et al., 2018), hands (Longo and Haggard, 2010; Longo, 2017), and the whole body (Fuentes et al., 2013a). Many studies demonstrated interesting distortions or plasticity of face, hands, and whole body topography in adults. In particular, several methods have been employed in studies examining face topography in adults; however, the methods used in these studies are not directly applicable to young children. For example, Mora et al. (2018) developed a proprioceptive pointing task to locate face landmarks in the first-person perspective. A vertical acrylic sheet was placed in front of the participants, very close to their face. Participants were asked to place their face on the chin rest and locate 11 face landmarks by finger pointing. Their findings suggested that size distortions are intrinsic to self-face representation. This task enables us to identify the features of face topography in adult participants; however, it is challenging to carry out the same task for young children because of difficulties such as providing verbal instructions and asking the children to maintain posture.

Our task was based on the mark test, a well-known test of mirror self-recognition (Gallup, 1970; Amsterdam, 1972), wherein children perform the required task without any instruction. However, while the mark test simply examines whether children recognize themselves in a mirror reflection, our task can additionally visualize their body topography in terms of their spatial error pattern and reaction time—quantifications that can be measured for children aged 2.5 and 3.5 years during the task repetition.

In this study, children aged 2.5 and 3.5 years were targeted because the ability to recognize oneself in a mirror reflection (mirror self-recognition) develops at approximately 24 months of age (Amsterdam, 1972; Nielsen and Dissanayake, 2004). Thus, evaluating face parts localization for children of these ages is important because their localization reflects the first organization of face topography. Among these, face topography is particularly noteworthy because we only see our faces reflected in the mirror. Therefore, correct localization of face parts may require matching proprioceptive and visual information for each face part. Since 2.5-year-olds are known to generally pass the mark test, we hypothesized that their initial face topography (without verbal instruction) could be examined using this AR task. We also hypothesized that the results of 2.5- and 3.5-year-olds could be compared quantitatively based on their developmental stages.

Taken together, the aim of this study was to develop a new task and assess its efficacy. We also examined whether this task would maintain children’s motivation for task repetition without difficult verbal instruction and whether it could be used to evaluate body topography in 2.5- and 3.5-year-olds.

## Experiment

### Participants

Forty-three 2.5-year-olds and 12 3.5-year-olds participated in this study. The final sample comprised 31 2.5-year-olds (15 females) and 11 3.5-year-olds (five females). Fourteen participants were excluded from the analysis (attrition rate = 25%) due to fussiness or embarrassment (*N* = 10), experimental error (*N* = 4), failure of the video recording, or because Kinect failed to properly identify their bones. The children were recruited from the participants’ pool of the NTT Communication Science Laboratories. This study was carried out in accordance with recommendations from the NTT Communication Science Laboratories Ethical Committee and with written informed consent from all participants’ parent(s). All parents gave written informed consent in accordance with the Declaration of Helsinki. The study protocol was approved by the NTT Communication Science Laboratories Ethical Committee.

### Apparatus and Task

**Figure 1:**
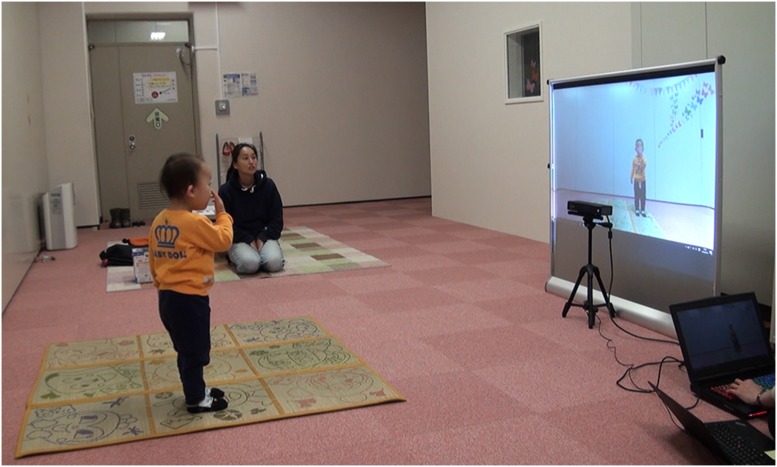
Experimental setup. Participants were asked to touch their real face with reference to their face image on projected digital images. If participants correctly touched the corresponding part of their real face, a cheerful visual and auditory reward was presented. To track participants’ face in 3D, a motion-sensing input device (Microsoft, Kinect v2) was used. The program to present digital images was written in Processing 3.0 and Kinect v2 for Processing library. We obtained permission from the participant’s parent for the publication of this image.

We developed a task called “Touching!” using a motion-sensing input device (Microsoft, Kinect v2) to track participants’ faces and using an AR technique to present participants with their projected self-image (EPSON, EB485WT) on the screen (KIMOTO, RUM60N1). Cartoon characters (digital image) then appeared on various parts of the children’s faces (nose, right/left cheek, lower/upper forehead, and chin; Figure 1). Participants’ bodies were presented in a mirror-like (ipsilateral) relationship. A motion-sensing input device was used to track participants’ faces in 3D. The program used to present digital images was written in Processing 3.0 and Kinect v2 for Processing library. We used a device with Graphics Processing Unit (GPU; DOSPARA, GALLERIA GAMEMASTER NX) for presenting the self-image with a maximum of 150 ms temporal delay (approximately four frames). A time delay of about 150 ms causes peculiarity, but it does not affect movement accuracy (Katayama et al., 2018). The movie *via* the USB camera of Kinect v2 was recorded by a monitor capturing device (Avermedia, AVT-C878) and laptop PC (Panasonic, CF-MX3).

Participants were asked to touch their real faces with reference to their face image on the projected digital images. First, a digital image was displayed on participants’ face part with a beep sound by the key input of the experimenter. This is the onset of each trial. If participants correctly touched the corresponding part of their real face, a cheerful visual and auditory reward was presented by the experimenter’s manual key press, and the digital image disappeared. If participants failed to touch, the digital image was displayed again on the same site but these responses were not used for analysis because such responses had considerable individual difference. If the experimenter judged that the participants had lost the motivation to touch, the trial was silently ended (the image disappeared), and the next trial began.

The first location presented by the digital image was always the nose because previous studies have shown that the nose is one of the first body parts children learn (Witt et al., 1990). The order of the following locations was randomized by Latin square design. In the experiment, to assess their motivation for task repetition, we prepared a relatively large number of trials: 37 trials maximum (1 example + 6 face places × 3 characters × 2 blocks).

To assess participants’ knowledge of vocabulary related to body parts, we asked their caregivers to complete a questionnaire, which included 60 words related to body parts (see Supplementary Table S1). Caregivers were requested to check whether their child could comprehend or comprehend and produce each word. To assess participants’ development of sensory profile, we asked the caregivers to answer the Japanese translation version of the Infant Toddler Sensory Profile (ITSP; Dunn and Daniels, 2002; Tsujii et al., 2015). The ITSP is a 48-item caregiver questionnaire that measures sensory modulation abilities as reflected in daily experiences in children aged 7 months to 36 months. Although ITSP is an assessment tool for evaluating sensory modulation behaviors in toddlers with autism spectrum disorders (ASDs), in this study, we used this tool to capture participants’ sensory modulation state only for exploring correlation with the task performance. We only asked the caregivers of 2.5-year-olds to answer the ITSP because the maximum age of eligibility for ITSP is 36 months (3 years). To assess participants’ experience of self-images in their daily life (mirror, video, pictures, etc.), we asked the caregivers how frequently the participants played with self-images and how they played with them.

### Procedure

To demonstrate the task rules, we first asked caregivers to play the “Touching!” game. The caregivers stood in front of the screen (height = 1.12 m) and the Kinect camera (height = 0.55 m from the ground, distance from participants’ position = 1.50 m; see Figure 1). Once the program was ready to capture their bones, the “Touching!” game began. They were asked to touch their real faces with reference to their face image on the projected digital image. At the beginning of the task, the caregivers demonstrated how to play the game and encouraged their children to participate in the game. A maximum of 13 trials were conducted during this pre-experiment phase. When the participants began to engage in the task spontaneously without caregiver’s guidance, we considered it as participants fully understanding the task and commenced the experiment. During the game, caregivers were asked not to say the names of body parts aloud because we wanted to assess body localization using visual and proprioceptive information and thus this instruction prevented the effect of semantic body knowledge.

After the practice phase by their caregivers, the children participated in the game. A total of 36 experimental trials were conducted (see “Task Repetition” section). The number of trials in the present experiment was based on those in previous studies (Witt et al., 1990; Brownell et al., 2010; Camões-Costa et al., 2011). In Brownell’s study, there were 12 trials for the task for 20- and 30-month-olds. In Witt et al. (1990) study, there were 20 trials for children aged 11–25 months. In Camoes-Costa’s study, 100 trials were conducted, although the participants were relatively older (age range: 26–41 months; mean age: 35 months). We considered more than 35 trials to be relatively high and thus, the experiment continued until the participants stopped participating.

### Analysis

The children’s responses were recorded in video clips. To analyze the error pattern and response time (RT) of their first touches, we coded participants’ correct/incorrect responses in each trial using frame by frame coding. The two coders were blind to the study’s goal. Error was defined as a failure of initial touch/pointing on the target body parts. Even if the participants correctly touched the target body part in their final touch after several explorations, we did not count such trials as correct. Body positions that the participants touched in error include mouth, eyes, temple, lip, neck, chest, etc. Inter-coder reliability based on correct/incorrect responses was calculated at 55% for all data. The coder agreement was *κ* = 0.88. The coders reached mutual agreement for the trials in which there were disagreements.

After discussing and seeking agreement in several cases, the second coder’s score was used. RT was defined as the duration between the appearance of the digital mark to participants’ first touch. In addition, we coded each trial for persistent response (more than two times repetitive touch across the trials), LR-error (left-right reversal error), and rear error (touch on the back of their head).

## Results

### Task Repetition

**Figure 2:**
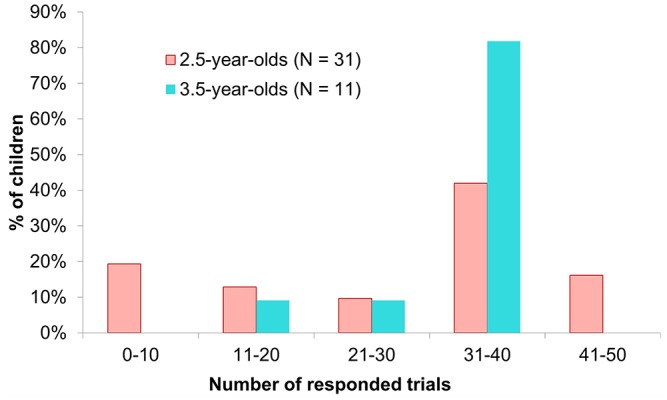
Number of executed trials. In this study, the maximum number of trials in the main experimental phase was 36. However, some 2.5-year-olds spontaneously participated in trials in the practice phase (Max. 12 trials). In such cases, we counted these trials the same as the main trials because these naive initial responses include important meanings for evaluating children’s body topography. Thus, some 2.5-year-olds were included in the bin of 41–50 trials.

To evaluate participants’ motivation for task repetition, we summarized the number of executed trials aggregately in Figure 2. More than 60% of the 2.5-year-olds executed more than 30 trials, while more than 80% of the 3.5-year-olds did so. Thus, both age groups maintained their motivation for the task despite the high level of repetition.

### Error Analysis

**Figure 3:**
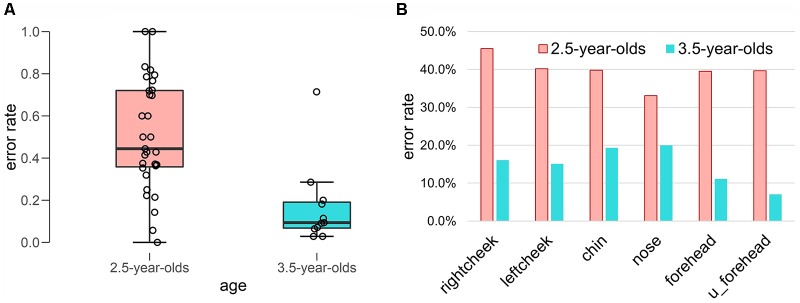
Error rate of initial body localization. **(A)** Box plot of error rate in each age group. **(B)** Error rate in each face position.

In Figure 3, the error rates are summarized in each age group panel Figure 3A and in each face position panel Figure 3B. In the 2.5-year-olds, the error rates varied widely, while in the 3.5-year-olds, they did not vary widely. A Welch-Satterthwaite *t*-test revealed a significant difference between the two age groups (*t*_(23.61)_ = 4.47, *p* < 0.001, Cohen’s *d* = 1.46).

To explore toddlers’ accuracy of face localization, we summarized the error rates for first touch in each face position (see Figure 3B) as follows: in the 2.5-year-olds, right cheek (45.5%), left cheek (40.2%), chin (39.8%), nose (33.1%), forehead (39.5%), and upper forehead (39.7%); in the 3.5-year-olds, right cheek (16.1%), left cheek (15.1%), chin (19.3%), nose (20.0%), forehead (11.1%), and upper forehead (7.0%). Although age differences were clear in all face positions, no clear differences were found between the error rates in each face position.

**Figure 4:**
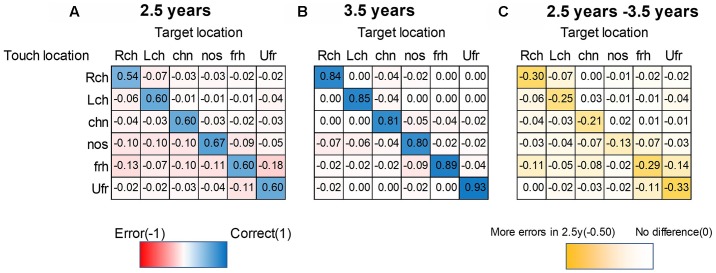
Heat map matrix between targeted- and touched-location in each age group. **(A)** 2.5-year-olds, **(B)** 3.5-year-olds. Blue-colored cells refer to correct touch rate (maximum correct: 1). Red cells refer to error rate (maximum error: −1). Yellow-colored cells in **(C)** refer to the subtraction of each rate between the two ages. Darker yellow indicates a larger difference between the two ages (maximum subtraction was −0.5 in this plot). The sum of absolute columns should be 1 (or less than 1, if other uninteresting parts like the foot are touched), while the sum of rows could exceed 1 since this matrix is asymmetric. For example, the nose was their favorite touched part regardless of the target (especially for 2.5-year-olds). Therefore, the sum of rows was more than 1 for the nose as the touched position, while the chin was not much preferred, and therefore, the sum of rows was less than 1. Note. Lch, left cheek; Rch, right cheek; Ufr, upper forehead; frh, forehead; nos, nose; chn, chin.

To analyze localization of initial touch in each age group and among the face positions, we summarized the heat map matrix in Figure 4 in each age group. Blue-colored cells refer to correct touch rate (maximum correct: 1.0). Red cells refer to error rate (maximum error: −1.0). Yellow-colored cells in (Figure 4C) refer to the subtraction of error between the two age groups. Darker yellow indicates a larger difference in error rate between the two age groups.

**Figure 5:**
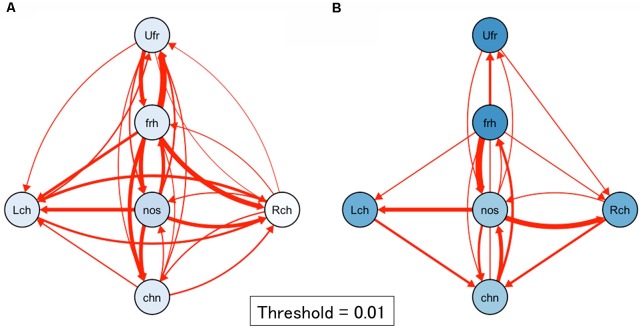
Network plots of first touch in each age group. **(A)** 2.5-year-olds, **(B)** 3.5-year-olds. Circles indicate each target face position. A bluer color refers to a higher rate of touching in initial touch (correct response). Red arrows indicate incorrect touch: the tips of the arrows indicate the error position and the bottom side of the arrows indicate the target positions. The thickness of the line with error rate values indicates frequency of error. A broader line indicates a higher frequency of error touch. In the 2.5-year-olds, the accuracy of localization was relatively low; localization errors varied both along the horizontal- and vertical-axes. In the 3.5-year-olds, the accuracy of localization became high; the variation of localization errors may be limited between adjacent parts. Note. Lch, left cheek, Rch, right cheek; Ufr, upper forehead; frh, forehead; nos, nose; chn, chin.

Next, to visualize the relationship among target positions and touched locations, we summarized Figure 4 into network plots (i.e., digraph) in Figure 5 in each age group. This visualization ascertained the initial touch pattern of each face position in each age group. Circles indicate each target face position and a cooler color refers to higher rate of touching in initial touch (correct response). Hotter arrows indicate incorrect touch, and the tops of the arrows indicate the error position while the bottom side of the arrows indicates the target position. Broader lines indicate higher frequency of error touch. In the 2.5-year-olds, the accuracy of localization was relatively low (i.e., the node colors are whiter); localization errors varied both along the horizontal- and vertical-axes. In the 3.5-year-olds, the accuracy of localization increased (i.e., the node colors are bluer); the variation of localization errors are limited between adjacent parts.

**Figure 6:**
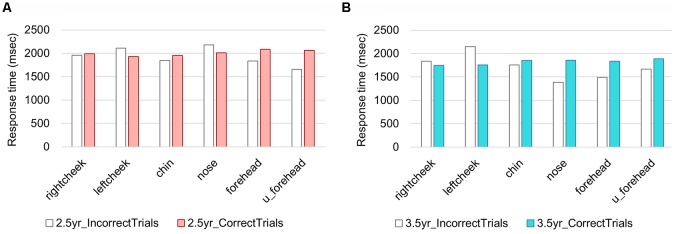
Response times (RTs) in each age group. **(A)** 2.5-year-olds, **(B)** 3.5-year-olds.

**Figure 7:**
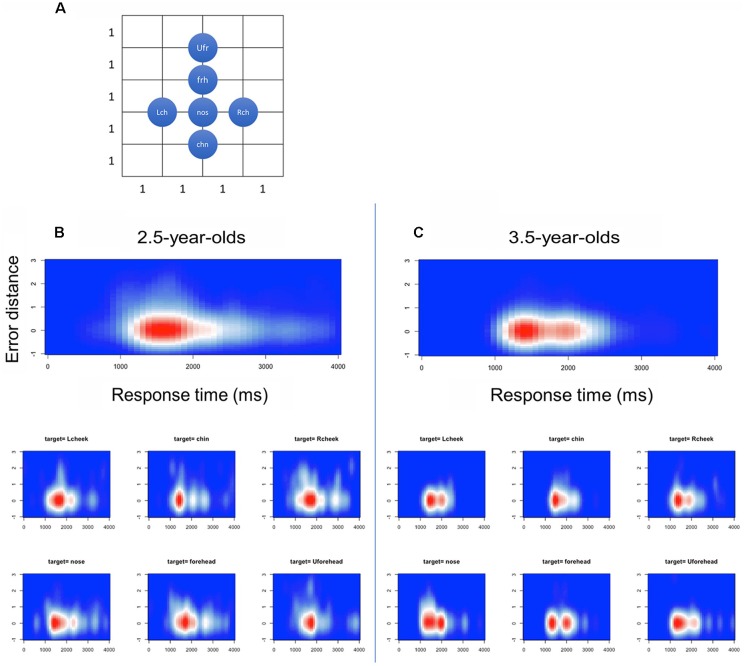
Heat maps in relation between reaction time (x) and error distance (y) in the first touch positions. **(A)** Definition of distance among the face parts, **(B)** 2.5-year-olds and **(C)** 3.5-year-olds.

In 2.5-year-olds, RT ranged from 33 ms to 177,100 ms. The median was 2,233 ms, while mode was 1,500 ms. To describe the distinctive features of the RT, we excluded the RTs that exceeded 4,000 ms (as a result, 83% of the overall data was included. This exclusion was made for RTs only). In Figure 6, mean RTs are summarized in correct/incorrect trials and in each target position. To capture the relationship among RTs, touch error, and age, we ascertain these relationships as heat maps in Figure 7. For the network analysis and heat map analysis, we summarized these error positions into the distances from the target positions (see Figure 7A). The x-axis refer to RTs, while the y-axis refers to the relative distance from each target position (see Figure 4 for the distance definition). For example, when the target is Rcheek (“Rch”) but the touched location is nose (“nos”), the distance is 1. When the target is Rcheek but the touched location is forehead (“frh”), the distance is 1.4 in Euclidean distance. The negative y is simply for visualization of the distribution tail. Therefore, the distance 0 indicates the correct responses. A hotter color refers to a higher frequency of responses. The initial touch responses to each target position in the 2.5-year-olds have a single peak, while responses to right/left cheek, nose, forehead, and upper-forehead in the 3.5-year-olds have a double peak. The spread of y-oriented error responses (refer to whiter colors) in each figure suggests localization errors. In general, variation of error position (y-axis) narrowed from the 2.5-year-olds to the 3.5-year-olds, as did the variation in reaction time (x-axis).

### Word Acquisition of Body Parts

In Table 1, the mean number of acquired words related to body parts was summarized in each age group. The number of acquired words (comprehension + production) was greater in the 3.5-year-olds (*M* = 47.1, *SD* = 10.5) than the 2.5-year-olds (*M* = 34.5, *SD* = 11.9), *t*_(38)_ = −3.08, *p* = 0.0001. The number of acquired words did not relate to the rates of initial touch errors in both age groups (*r* = −0.274, *p* = 0.087). The acquired rate of each target face position word was as follows: in the 2.5-year-olds, cheek (90%), forehead (59%), nose (97%), and chin (38%); in the 3.5-year-olds, cheek (100%), forehead (100%), nose (100%), and chin (73%).

**Table 1:** Mean number of lexical acquisition of body parts (60 words) and mean acquisition rate of the target position based on caregiver report.

	*n*	*M*	*SD*	Cheek	Forehead	Nose	Chin
2.5-year-olds							
Comprehension	29	11.72	7.24	97%	93%	100%	62%
Comp+Production	29	34.52	11.86	90%	59%	97%	38%
3.5-year-olds							
Comprehension	11	10.55	7.16	100%	100%	100%	91%
Comp+Production	11	47.09	10.50	100%	100%	100%	73%

*Note. Two of the 2.5-year-olds were excluded from the analysis due to data unavailability (*N* = 2)*.

### Sensory Profile [Japanese Translation Version of Infant Toddler Sensory Profile (ITSP)]

We explored the relationship between the task performance and sensory profile in the 2.5-year-olds, excepting the 3.5-year-olds because the ITSP is only suitable up to 36 months. We calculated Spearman’s rank correlation coefficient among the task performances (number of correct/incorrect trials, error rate), sensory profile (auditory, visual, tactile, vestibular, and oral sensory), and sensory types (low registration, sensation seeking, sensory sensitivity, and sensation avoiding; see Supplementary Table S2). No significant correlations were found between the task performance and sensory profile.

### Participants’ Experience of Play Using Self-Images

To analyze the relationship between frequencies of play using self-image and the accuracy of body localization, we collected data of frequency of play using self-images (see camera, video, mirror, cell phone applications using self-images). The frequencies of play using self-image did not relate to the performance of body localization in either age group (2.5-year-olds: ρ_(*n* = 31)_ = 0.058, *p* = 0.760; 3.5-year-olds: ρ_(*n* = 11)_ = −0.052, *p* = 0.880).

## Summary and Future Directions

In this study, we developed a new face localization task to overcome the issue of maintaining young children’s motivation for task repetition. Using the AR technique and 3D face-tracking technology, we presented 2.5- and 3.5-year-olds with their projected self-image on a screen accompanied by a digital mark located on positions of their face, and then required them to touch these marks on their own bodies. Nearly half of 2.5-year-olds repeatedly executed more than 30 trials and almost all 3.5-year-olds executed all 36 trials.

Although the body localization tasks used in previous studies may underestimate young children’s knowledge of body structure due to the high level of repetition without any reward (Brownell et al., 2010, 2012), our new task was able to maintain young children’s motivation for task repetition. Our task is also expected to reveal the details of young children’s face topography because repetitive data collection for the same face parts enables us to calculate the error rate in each face position. Furthermore, the analyses, which comprised combined multiple measurements such as error position of initial touch, relative distance from the target position, and RT, helped us to reveal the characteristics of face topography in young children.

In the present study, we found a clear age contrast for localization accuracy between 2.5- and 3.5-year-olds. For example, errored positions were broader in the 2.5-year-olds than in the 3.5-year-olds. This finding suggests that face topography in 2.5-year-olds is relatively more blurry than in 3.5-year-olds. From analysis of the RTs, we also captured the difference between the two age groups. The RTs of initial touch in the 2.5-year-olds showed a single peak, while the responses to right/left cheek, nose, forehead, and upper-forehead in the 3.5-year-olds showed a double peak. It is likely that 2.5-year-olds’ touch demonstrates ballistic touching, which is relatively fast, a straight path, and without adjustment, while 3.5-year-olds’ touch includes visual proprioceptive motor control as well as ballistic touching. Ballistic touching might reflect proprioceptive localization of face parts; therefore, the children, particularly the 2.5-year-olds, demonstrated incorrect touch without modification of their initial touch. On the other hand, 3.5-year-olds showed touches with relatively longer RTs. This might include visual-proprioceptive motor control with reference to visual feedback from own hands on the screen; therefore, incorrect localizations might be modified by the way of touching.

How can the findings of the present study be considered in adult studies of face topography? As stated above, in Mora et al. (2018) study, adult participants were asked to point to 11 face landmarks (i.e., hairline, corners of each eye, tip of nose, lateral side of both nostrils, corners of the mouth, and chin). The results showed overestimated of the width of the nose and mouth (Mora et al., 2018). This is the first task to evaluate proprioceptive-based face topography in adults. The participants were asked to point to their face parts according to the verbal instruction without visual feedback. However, it is difficult to carry out the same task among young infants. In this study, we used a mirror test as a hint to develop a pointing task with visual feedback. By introducing visual feedback and enabling pointing by visuo-motor control, purely proprioceptive-based face topography cannot be evaluated; however, the main research question in the present study was whether young children can maintain their motivation for task repetition. Therefore, a direct comparison with the Mora et al. (2018) findings is a task for the future. However, if we can sophisticate our task to distinguish visuo-motor control and measure the mistouched points in more detail, we will be able to quantify the distortion of the face topography.

In recent years, research on the plasticity of the face representation, known as the enfacement illusion (Sforza et al., 2010), has also attracted attention (Porciello et al., 2018 for a review; Serino et al., 2015; Estudillo and Bindemann, 2017). Similar to the rubber hand illusion (RHI; Botvinick and Cohen, 1998) and the out-of-body illusion (Ehrsson, 2007), it is an approach to examine the plasticity of self-face representation using the self-other discrimination task by controlling multisensory stimulation between the self and others. The boundaries of self-other distinctions might be more ambiguous in young children than in adults, and several theories suggest that the state of undifferentiated self and-other promotes sociality (the like-me theory; Meltzoff, 2007; the social-biofeedback model; Gergely and Watson, 1999). Based on these perspectives, it is intriguing to examine the likelihood and development of the enfacement illusion in young children. In particular, it is worth noting whether to remap tactile/motor information, or whether it can be initially processed in a supramodal manner.

What advantage does the “Touching!” task have for examining the development of body recognition? Before discussing this issue, let us introduce three types of body representation as proposed by Schwoebel and Coslett (2005). The first, termed the body schema, comprises on-line sensorimotor representation of the body. Actual and mentally simulated movements depend on the body schema; this can be estimated by the hand imagery/action task (Sirigu et al., 1996) and the hand laterality task (Parsons et al., 1995). The second type, termed body topography, comprises a topological map of the body. This does not require verbal knowledge of the body. A typical example of impairment of body topography is autotopagnosia, which is characterized by an inability to localize body parts on one’s own or others’ bodies (Buxbaum and Coslett, 2001). Body topography has two types of representation: one based on tactile sensation and the other based on proprioceptive sensation. The third, termed body image, comprises a semantic and lexical representation of the body. Following an examination among brain-injured patients, Schwoebel and Coslett proposed these putative three types of body representation that assume independent neural pathways.

The developmental transitions of tactile-based body topography during the first year of life emerge consecutively (Somogyi et al., 2018; Meltzoff et al., 2018, 2019). At the neural level, the somatosensory brain map differentiates from early in life. Meltzoff, Saby, and Marchall examined the neural representation of the body in 60-day-old human infants. Electroencephalography (EEG) was recorded while infants received tactile stimulation of three body parts: hand, foot, and lip. Tactile stimulation of these body parts elicited distinguishable signatures (Meltzoff et al., 2019). Interestingly, however, concerning aspects of body localization, infants do not touch their hand(s) to the correct body positions until 7.5-months-old (Somogyi et al., 2018). Somogyi et al. (2018) examined the ability for body localization during the first months of life by examining localization of vibrotactile stimulation on infants’ limbs. The dissociation between neural differentiation of tactile sensation and practical use in localization is interesting and important to reveal the developmental transition of body topography.

In this study, we assume that “Touching!” can be used to evaluate body topography based on proprioceptive sensation because the participants could not find projected marks without using proprioceptive information related to their body. We also found that accuracy of face localization did not correlate with the word acquisition of body parts. This finding supports the fact that acquisition of body topography and the emergence of semantic and lexical knowledge of the body are independent of each other. Further research is necessary, however, to confirm this finding.

Considering the development of proprioceptive body topography, few studies have examined this topic in young children. Most relevant literature on this topic examines the RHI in childhood, which is a famous experimental paradigm to reveal the nature of body ownership. The subjective sense of body ownership is constructed by multimodal integration among visual, proprioceptive, and tactile information (Botvinick and Cohen, 1998). Recent works suggest that there are two dissociable processes of body representation; a process based on visual-tactile information and a later-maturing process based on visual-proprioceptive information (Biko et al., 2012; Cowie et al., 2013). These examinations using the RHI are helpful to clarify the cognitive background of body ownership based on visual-proprioceptive information, whereas it would be too difficult for children under four to complete the RHI task due to the difficulty of verbal instruction.

Our task does not require the report of illusion or an understanding of complex verbal instruction. Therefore, it is helpful for examining the development of proprioceptive face topography in very young children.

This study has several limitations. The first limitation is related to face and bone detection. In this study, we used Microsoft Kinect for the detection of face and bones. However, the face and bone model used for basic programming might be adjusted for adults’ size. Thus, the face and bone detection were sometimes off the correct position because toddlers are small and have short limbs. Nevertheless, the current system could detect the face and bones correctly in most trials. A body localization task should be developed in further research using other detection devices. The second limitation is also related to face and bone detection. There were cases in which bone detection was difficult due to the temperament of the participants. For example, some children have a strong bonding need and cannot stay away from their mothers. Thus, sampling bias is inevitable. If there is a technique to detect the bone even if the child is near their mother, this limitation can be overcome to some extent. The third limitation is that there are several possible explanations for the touching. One possibility is that the development of the proprioceptive face topography affects touch, while another possibility is that simple visuo-motor control without the knowledge of self-face affects touch. We consider that the former is highly possible because differences in reaction patterns are seen in each digital image presentation location, but it is difficult to completely separate these two possibilities in this study. In future, it is necessary to compare tasks that can distinguish visuo-motor control, such as tasks that involve touching of one’s own body parts and tasks that involve touching the parts of a toy.

In future research, we would like to extend the “Touching!” task to estimate whole-body localization. Furthermore, it is also important to reveal the developmental transition and relationship between the tactile and proprioceptive body topographies.

## Data Availability

The datasets generated for this study are available on request to the corresponding author.

## Ethics Statement

This study was carried out in accordance with the recommendations of the NTT Communication Science Laboratories Ethical Committee with written informed consent from all subjects. All subjects gave written informed consent in accordance with the Declaration of Helsinki. The protocol was approved by the NTT Communication Science Laboratories Ethical Committee.

## Author Contributions

MM, RM, and TA conceptualized, designed the study and analyzed the data and the results were interpreted and examined by all authors. MM and RM collected the data. MM wrote the first draft of the manuscript. All authors agreed on the final version of the manuscript.

## Conflict of Interest Statement

MM was an unpaid guest researcher at NTT Communication Science Laboratories and RM was employed as a research scientist by NTT Communication Science Laboratories. The remaining author declares that the research was conducted in the absence of any commercial or financial relationships that could be construed as a potential conflict of interest.
