# Corrigendum: Touching! An Augmented Reality System for Unveiling Face Topography in Very Young Children

**DOI:** 10.3389/fnhum.2019.00236

**Published:** 2019-07-12

**Authors:** Michiko Miyazaki, Tomohisa Asai, Ryoko Mugitani

**Affiliations:** ^1^Department of Social Information Studies, Otsuma Women's University, Tokyo, Japan; ^2^NTT Communication Science Laboratories, Atsugi, Japan; ^3^Advanced Telecommunications Research Institute International (ATR), Kyoto, Japan; ^4^The Faculty of Integrated Arts and Social Sciences, Japan Women's University, Kanagawa, Japan

**Keywords:** psychology, development, body image, augmented reality, face, body topography

In the Acknowledgments section of the original article, the names of two individuals were spelled incorrectly. The first error is **Ayano Shiina**; the correct spelling is **Ayana Shiino**. The second error is **Koichi Matsuda**; the correct spelling is **Kouichi Matsuda**.

The authors apologize for the oversight and confirm that this does not change the scientific conclusions of the article in any way. The original article has been updated.

