# Supplementary material for: Touching! An Augmented Reality System for Unveiling Face Topography in Very Young Children
Source: Front Hum Neurosci. 2019 Jun 11;13:189. doi: 10.3389/fnhum.2019.00189 (PMC6579857; doi:10.3389/fnhum.2019.00189)
Supplement: Supplementary file 1 [file Table_1.docx]

Supplementary Material

Touching! An augmented reality system for unveiling
face topography in very young children

Michiko Miyazaki*, Tomohisa Asai, and Ryoko Mugitani

*** Correspondence:** Michiko Miyazaki: myzk@otsuma.ac.jp

# Supplementary Figures and Tables

## Supplementary Tables

**Supplementary Table 1.** Mean acquisition rates of body-related words (60 words) based on caregiver report.

|  | Cheek | Forehead | Nose | Chin | Body | Naked | Skin | Head | Hair | Down | Eyebrow | Eyelash | Eyelid | Eye | Ear |
| --- | --- | --- | --- | --- | --- | --- | --- | --- | --- | --- | --- | --- | --- | --- | --- |
| 2.5-yr |  |  |  |  |  |  |  |  |  |  |  |  |  |  |  |
| Comprehension | 97% | 93% | 100% | 62% | 79% | 79% | 45% | 100% | 97% | 62% | 59% | 34% | 21% | 100% | 93% |
| Comp+Expression | 90% | 59% | 97% | 38% | 45% | 59% | 3% | 86% | 69% | 21% | 28% | 10% | 0% | 97% | 93% |
| 3.5-yr |  |  |  |  |  |  |  |  |  |  |  |  |  |  |  |
| Comprehension | 100% | 100% | 100% | 91% | 82% | 91% | 64% | 100% | 100% | 82% | 82% | 55% | 45% | 100% | 100% |
| Comp+Expression | 100% | 100% | 100% | 73% | 82% | 82% | 36% | 100% | 91% | 55% | 55% | 27% | 27% | 100% | 100% |

|  | Mouth | Lip | Tongue | Beard | Tooth | Bad tooth | Face | Throat | Neck | Shoulder | Back | Breast | Nipple | Stomach |
| --- | --- | --- | --- | --- | --- | --- | --- | --- | --- | --- | --- | --- | --- | --- |
| 2.5-yr |  |  |  |  |  |  |  |  |  |  |  |  |  |  |
| Comprehension | 100% | 34% | 52% | 52% | 100% | 48% | 100% | 31% | 69% | 72% | 79% | 90% | 17% | 93% |
| Comp+Expression | 90% | 10% | 28% | 24% | 79% | 21% | 79% | 14% | 45% | 41% | 38% | 79% | 0% | 86% |
| 3.5-yr |  |  |  |  |  |  |  |  |  |  |  |  |  |  |
| Comprehension | 100% | 64% | 82% | 82% | 100% | 100% | 100% | 82% | 91% | 100% | 91% | 100% | 45% | 100% |
| Comp+Expression | 100% | 18% | 55% | 64% | 100% | 64% | 100% | 64% | 82% | 73% | 64% | 100% | 9% | 100% |

|  | Navel | Armpit | Arm | Elbow | Nail | Hand | Wrist | Finger | Thumb | Index finger | Middle finger | Third finger | Pinkie | Hips |
| --- | --- | --- | --- | --- | --- | --- | --- | --- | --- | --- | --- | --- | --- | --- |
| 2.5-yr |  |  |  |  |  |  |  |  |  |  |  |  |  |  |
| Comprehension | 86% | 41% | 79% | 31% | 83% | 100% | 21% | 76% | 28% | 24% | 14% | 17% | 17% | 97% |
| Comp+Expression | 72% | 14% | 28% | 17% | 69% | 97% | 7% | 55% | 0% | 0% | 0% | 0% | 0% | 90% |
| 3.5-yr |  |  |  |  |  |  |  |  |  |  |  |  |  |  |
| Comprehension | 91% | 55% | 91% | 64% | 100% | 100% | 36% | 100% | 55% | 45% | 36% | 36% | 55% | 100% |
| Comp+Expression | 82% | 36% | 82% | 45% | 91% | 100% | 9% | 82% | 27% | 18% | 18% | 18% | 18% | 100% |

|  | Foot | Thigh | Knee | Sole | Ankle | Heel | Toe | Mole | Bone | Blood | Beak | Tail | Wing | Horn |
| --- | --- | --- | --- | --- | --- | --- | --- | --- | --- | --- | --- | --- | --- | --- |
| 2.5-yr |  |  |  |  |  |  |  |  |  |  |  |  |  |  |
| Comprehension | 97% | 14% | 41% | 41% | 17% | 24% | 28% | 24% | 10% | 55% | 14% | 79% | 38% | 17% |
| Comp+Expression | 90% | 7% | 28% | 10% | 3% | 10% | 3% | 14% | 3% | 28% | 3% | 55% | 17% | 7% |
| 3.5-yr |  |  |  |  |  |  |  |  |  |  |  |  |  |  |
| Comprehension | 100% | 36% | 82% | 64% | 27% | 55% | 45% | 64% | 73% | 91% | 55% | 100% | 91% | 91% |
| Comp+Expression | 100% | 9% | 55% | 36% | 9% | 9% | 9% | 45% | 36% | 82% | 27% | 91% | 64% | 55% |

**Supplementary Table 2.** Spearman’s rank correlation coefficient among task performance (number of correct/incorrect trials, error rate), each sensory profile (auditory, visual, tactile, vestibular, oral sensory), and sensory types (low registration, sensation seeking, sensory sensitivity, and sensation avoiding). Bold indicates statistically significant correlation. ** *p* < .01, * *p* < .05.

|  | Incorrect Trials | Correct Trials | TotalResponsedTrial | Error Rate | Auditory | Visual | Tactile | Vestibular | Oral | LowRegistration | Sensation Seeking | Sensory Sensitivity | Sensation Avoiding |
| --- | --- | --- | --- | --- | --- | --- | --- | --- | --- | --- | --- | --- | --- |
| Incorrect Trials | 1 |  |  |  |  |  |  |  |  |  |  |  |  |
| Correct Trials | -0.012 | 1 |  |  |  |  |  |  |  |  |  |  |  |
| TotalResponsedTrial | **.553^**^** | **.826^**^** | 1 |  |  |  |  |  |  |  |  |  |  |
| ErrorRate | **.362^*^** | **-.739^**^** | **-.412^*^** | 1 |  |  |  |  |  |  |  |  |  |
| Auditory | -0.033 | -0.078 | -0.084 | -0.101 | 1 |  |  |  |  |  |  |  |  |
| Visual | -0.144 | -0.177 | -0.229 | 0.049 | **.392^*^** | 1 |  |  |  |  |  |  |  |
| Tactile | -0.178 | -0.036 | -0.130 | -0.057 | **.749^**^** | 0.347 | 1 |  |  |  |  |  |  |
| Vestibular | -0.023 | 0.080 | 0.054 | -0.048 | **.558^**^** | **.449^*^** | **.592^**^** | 1 |  |  |  |  |  |
| Oral | -0.284 | 0.082 | -0.091 | -0.264 | **.710^**^** | 0.330 | **.783^**^** | **.528^**^** | 1 |  |  |  |  |
| LowRegistration | -0.136 | -0.133 | -0.188 | -0.017 | **.892^**^** | **.400^*^** | **.782^**^** | **.569^**^** | **.703^**^** | 1 |  |  |  |
| Sensation Seeking | -0.083 | 0.101 | 0.038 | -0.289 | **.631^**^** | **.682^**^** | **.505^**^** | 0.308 | **.539^**^** | **.512^**^** | 1 |  |  |
| Sensory Sensitivity | -0.141 | 0.041 | -0.045 | -0.050 | **.635^**^** | 0.200 | **.772^**^** | **.724^**^** | **.659^**^** | **.624^**^** | 0.106 | 1 |  |
| Sensation Avoiding | -0.088 | -0.185 | -0.204 | 0.127 | **.626^**^** | 0.333 | **.830^**^** | **.730^**^** | **.742^**^** | **.623^**^** | 0.223 | **.827^**^** | 1 |
